# The value of midwifery counseling during lactation as a key factor of pharmacovigilance

**DOI:** 10.18332/ejm/156439

**Published:** 2022-11-24

**Authors:** Maria Tigka, Victoria Vivilaki, Dimitra Metallinou, Christina Nanou, Aikaterini Lykeridou

**Affiliations:** 1 Department of Midwifery, School of Health and Care Sciences, University of West Attica, Athens, Greece; 2 Obstetric Emergency Department, General and Maternity Hospital ‘Helena Venizelou’, Athens, Greece

**Keywords:** midwifery, lactation, counseling, medication use, pharmacovigilance

Breastfeeding has proven benefits for infants, including reduced morbidity and mortality, reduced acquired infections and a positive impact on cognitive functions^1^. At present, the reported initiation of breastfeeding reaches a rate of 90% in some European countries, but exposure to pharmaceuticals in the postpartum period varies widely from 34% to 100%^2^. Multiple conditions (chronic or acute) requiring pharmacological treatment may arise during breastfeeding. Decisions about pharmaceutical therapy require consideration regarding the effect on the mother and her child^3^. Unfortunately, the need for pharmaceutical therapy during lactation has led many women to discontinue breastfeeding either due to unclear counseling by healthcare professionals (HPs) or fear of harming their child^2-4^.

Currently, the quality of information on the safety of certain pharmaceutical products during lactation varies and recommendations for the use of medicines during lactation are inconsistent. This may affect the ability of HPs to convey information on drug safety to breastfeeding women and may lead to inappropriate advice for breastfeeding cessation and complexity in the decision-making process^5^. Previous studies on physicians’ knowledge about medication safety during lactation have disappointingly shown that approximately 53% of the participants were unaware that antiepileptic drugs are acceptable during lactation and mothers can breastfeed safely^6^. Additionally, the 39% of them believed that breastfeeding is contraindicated in women with prescribed antidepressant drug therapy^7^. Conflicting information about pharmaceutical products safety during lactation and lack of counseling skills are important barriers for the initiation and duration of breastfeeding and, therefore, emphasis should be given on the promotion of pharmacovigilance in pregnant and lactating mothers.

There are limited data to support clinical decisions in the perinatal period due to the fact that it is difficult to recruit breastfeeding women and their infants in clinical trials as they are considered vulnerable^8,9^. The ethical considerations that may arise in such cases are whether a breastfeeding mother should use a medication for which data are lacking and expose her infant to potential risk, or discontinue breastfeeding for the duration of the study so that the infant is not exposed to the investigational medicine^10,11^. Furthermore, pharmaceutical industries are discouraged from involving breastfeeding mothers in clinical trials due to possible litigation and relatively little benefit compared to the investment; their inclusion requires long-term follow-up in order to assess infant outcomes^10^. To enable the safe use of pharmaceuticals during lactation, alternative methods have been developed to overcome the derived barriers. Population pharmacokinetic (pop PK) modeling, for example, provides evaluations on infant drug exposure via breast milk through simulation. Preliminary results obtained from studies using pop PK modeling enhance the validity of the model-based simulations^12-14^.

Pharmacovigilance procedures in the European Union regarding the use of medicines during lactation is also an area that needs further guidance. Once a pharmaceutical product is placed on the market, it is important that data are collected to better understand the risks associated with its use. Pharmacokinetic data in an infant after exposure to a medicine through breast milk provide important information on the potential risks for a nursing child, and when an adverse effect is suspected, it may be valuable to obtain a blood sample from the child for further analysis. Spontaneous reports during the post-authorization phase are a critical source of information on adverse effects occurring during lactation^9^. There is an advancing interest on pharmacovigilance during perinatal period by midwives and other HPs^15-17^. However, to our knowledge, only one study has been conducted on pharmacovigilance in the postnatal period^18^ which describes spontaneous reports of adverse drug reactions (ADRs) of drugs transmitted via breast milk based on data included in the French pharmacovigilance database. The ADRs were reported by HPs in 88.7% of the cases and the rest by individuals. The most commonly reported ADRs were neurological and gastrointestinal, and suspected medicines included antiepileptics, opioid analgesics and benzodiazepines.

Community midwifery could contribute to pharmacovigilance systems and detect safety signals from the use of medicines during the perinatal period^19^. European legislation on the safety of medicines should lead to regulatory turning points and provide an additional level of public health protection from potential harm to breastfeeding mothers and their infants, caused by maternal medication intake^20^. Therefore, midwives could play an active role in the detection, evaluation, understanding, and prevention of ADRs or any other drug-related health problem during lactation^9^. This important action research should focus on reporting information on a possible causal relationship between an adverse event and a drug deriving from a variety of sources rather than individual case reports of mother-child dyads, where causality can be ambiguous^20^. Risk–benefit analysis during lactation mainly refers to the systematic collection of information in order to clarify suspicious events while using a pharmaceutical product and also to identify medicine’s risks and benefits. Increased and adequate data collection and their timely evaluation will allow mothers and midwives to have relevant information to make informed decisions about the use of medicines during lactation and will provide the scientific committee and regulatory authorities with essential information to update healthcare databases^11^. Neither continuation nor discontinuation of medicines is without risk, but harm can be minimized by effective pharmacovigilance^21^.

## Data Availability

Data sharing is not applicable to this article as no new data were created.
